# Consumption of a high energy density diet triggers microbiota dysbiosis, hepatic lipidosis, and microglia activation in the nucleus of the solitary tract in rats

**DOI:** 10.1038/s41387-020-0119-4

**Published:** 2020-06-09

**Authors:** Dulce M. Minaya, Anna Turlej, Abhinav Joshi, Tamas Nagy, Noah Weinstein, Patricia DiLorenzo, Andras Hajnal, Krzysztof Czaja

**Affiliations:** 1 Department of Veterinary Biosciences and Diagnostic Imaging, University of Georgia, Athens, GA 30602 United States; 2 Department of Pathology, University of Georgia, Athens, GA 30602 United States; 3 Department of Psychology, Binghamton University, Binghamton, NY 13902 United States; 4 Department of Neural and Behavioral Sciences, the Pennsylvania State University, College of Medicine, Hershey, PA 17033 United States

**Keywords:** Feeding behaviour, Neuroscience, Obesity

## Abstract

**Introduction:**

Obesity is a multifactorial chronic inflammatory disease. Consumption of high energy density (HED) diets is associated with hyperphagia, increased body weight and body fat accumulation, and obesity. Our lab has previously shown that short-term (4 weeks) consumption of a HED diet triggers gut microbiota dysbiosis, gut inflammation, and reorganization of the gut-brain vagal communication.

**Objetives:**

The aim of this study was to investigate the effect of long-term (6 months) consumption of HED diet on body composition, gut microbiome, hepatocellular lipidosis, microglia activation in the nucleus of the solitary tract, and systemic inflammation.

**Methods:**

Male Sprague–Dawley rats were fed a low energy density (LED) diet for 2 weeks and then switched to a HED diet for 26 weeks. Twenty-four-hour food intake, body weight, and body composition were measured twice a week. Blood serum and fecal samples were collected at baseline, 1, 4, 8, and 26 weeks after introduction of the HED diet. Serum samples were used to measure insulin, leptin, and inflammatory cytokines using Enzyme-linked Immunosorbent Assay. Fecal samples were assessed for 16 S rRNA genome sequencing.

**Results:**

HED diet induced microbiota dysbiosis within a week of introducing the diet. In addition, there was significant microglia activation in the intermediate NTS and marked hepatic lipidosis after 4 weeks of HED diet. We further observed changes in the serum cytokine profile after 26 weeks of HED feeding.

**Conclusions:**

These data suggest that microbiota dysbiosis is the first response of the organism to HED diets, followed by increased liver fat accumulation, microglia activation in the brain, and circulating levels of inflammatory markers. To our knowledge, this is the first study to present longitudinal and cross-sectional results on effect of long-term consumption of HED diets on all these parameters in a single cohort of animals.

## Introduction

Obesity is a low-grade, chronic inflammatory disease. However, the relationship between inflammation and obesity, and the factors behind obesity-dependent inflammation are not well understood. Typically, inflammation is a transient physiological response. However, the inflammatory state that accompanies the metabolic syndrome observed in most obese individuals is not transient.

Consumption of high energy density (HED) diets triggers microbiota dysbiosis, increased body fat accumulation, and metabolic syndrome^1,2^. The vast majority of obesity cases result from an imbalance between energy intake and energy expenditure, with intake surpassing expenditure. Excess energy intake leads to enlargement of adipose tissue depositions. Once thought to be inert, adipose tissue is now recognized as a metabolically active endocrine organ^3^. This enlargement occurs through an increase in the number of adipocytes (adipogenesis) or an increase in the size of existing adipocytes (hypertrophy)^4^. In the *db/db* mouse model, there is a high number of adipogenic/angiogenic cell clusters in the early stages of obesity. The number of these cell clusters declines over time and there is an increase in the number of crown-like structures, which are hallmarks of local infiltration of macrophages into tissue surrounding dead adipocytes^5^. Using three independent adipocyte-specific anti-inflammatory mouse models, Asterholm et al. showed that an acute inflammatory response in adipose tissue is necessary to stimulate adipogenesis as well as proper remodeling and angiogenesis of the extracellular matrix, to allow for healthy adipose tissue expansion^6^. It would appear that the tonic activation of the innate immune system induced by excess energy intake gradually disrupts the homeostatic state, triggering chronic inflammation.

In the obese state, the production of proinflammatory adipokines induce resident macrophages to change their phenotype from surveillance “M2” to proinflammatory “M1” as well as trigger recruitment of M1 macrophages^7,8^. In addition, free fatty acids (FFAs) activate toll-like receptor (TLR) 4 in adipose tissue to generate proinflammatory signals^9,10^. Moreover, deletion of TLR5 triggered a shift in the species composition of the gut microbiota that is associated with development of metabolic syndrome^11^. Previous work from our laboratory has shown that 4 weeks of HED diet is sufficient to trigger microglia activation in the nucleus of the solitary tract (NTS)^12^.

Another obesity comorbidity is non-alcoholic fatty liver disease (NAFLD), the most common chronic liver condition in the Western world^13^. NAFLD is found among people with diabetes (50%) and obesity (76%), and it is almost universal among diabetic people who are morbidly obese^14^. It encompasses a wide spectrum of liver damage that occurs in people who drink little to no alcohol. Hepatocytes play a primary role in lipid metabolism. FFAs enter the hepatocyte and most FFAs are esterified to form triglycerides (TGs). TGs form complexes with an apolipoprotein to form lipoproteins and then are exported from the hepatocyte. The apolipoproteins are synthesized by the hepatocyte and this is the rate-limiting step in TG export. When consuming a HED diet, the usual cause of hepatic lipidosis is the increased production of TGs, which outpaces apolipoprotein production^15^.

The aim of this study was to investigate the systemic responses to long-term consumption of a HED diet. Our main goal was to determine the timeline progression of the systemic changes—increased body fat accumulation, development of microbiota dysbiosis, changes in serum of cytokines, development of NAFLD, and increased microglia activation—induced by HED diet consumption. We tested the hypotheses that HED diet consumption induces progressive microbiota dysbiosis and increases circulating levels of leptin, insulin, and proinflammatory cytokines. We also hypothesized that HED diet consumption induces NAFLD and increases microglia activation in the NTS.

## Methods

### Animals

Male Sprague–Dawley rats (*n* = 15; ~300 g; Envigo, Indianapolis, IN) were housed individually in conventional polycarbonate shoe-box cages in a temperature-controlled vivarium with ad libitum access to low energy density (LED; 5% fat, 3.25% sucrose) pellets of rat chow (PicoLab rodent diet 20, product #5053, Fort Worth, TX) and water. Rats were maintained on a 12:12-h light: dark cycle with lights on at 0700-h and allowed to acclimate to laboratory conditions for 1 week prior to starting experiments. All animal procedures were approved by the University of Georgia Institutional Animal Care and Use Committee and conformed to National Institutes of Health Guidelines for the Care and Use of Laboratory Animals.

### Food Intake, body weight, and body composition

Following the acclimation period, rats were maintained on LED for an additional 2 weeks and were then switched to a HED diet (45% fat, 20% sucrose, Research Diet #D12451, New Brunswick, NJ). Food intake was measured twice a week as previously reported^16^. Body weight and body composition were measured weekly using a minispec LF 110 BCA Analyzer (Bruker Corp., The Woodlands, TX). Six rats, chosen randomly, were sacrificed after being on HED diet for 4 weeks (ST-HED, Supplementary Fig. S1). The remaining nine rats were maintained on HED diet for a total of 26 weeks (LT-HED, Supplementary Fig. S1). An additional aged-matched, LED fed group of rats (*n* = 9, LED26, Supplementary Fig. S1) served as the endpoint controls for the LT-HED group.

### Cytokines, leptin, and insulin levels in serum

Blood samples were collected on the last day of LED and 4, 8, and 26 weeks after introduction of the HED diet. The serum was collected and stored at −21 °C. A cytokine array (Rat Cytokine ELISA Kit, cat #EA-4006, Signosis Inc., Sunnyvale, CA) was used to measure levels of cytokines and chemokines. Insulin levels were determined using the Rat Insulin ELISA kit (cat #80-INSRT-E01; ALPCO Diagnostics, Inc., Salem, NH).

### Microbiome analysis

Fecal samples were collected following the same timeline as for blood samples mentioned above. Bacterial DNA was extracted from feces using a commercial kit (Quick-DNA Fecal/Soil Microbe Miniprep Kit, cat #D6010, Zymo research, Irvine, CA). High-throughput sequencing was performed using Illumina MiSeq paired-end runs (GGBC, Athens, GA). Amplification targeted the V3–V4 region of the 16 S ribosomal RNA genes using the following primers: S-D-Bact-041-b-S-17 (5’-CCTACGGGNGGCWGCAG-3’) forward and S-D-Bact-0785-a-A-21 (5’-GACTACHVGGGTATCTAATCC-3’)^17^. Sequences were subsequently trimmed, joined, and quality filtered. To identify Operational Taxonomic Units (OTUs) and to evaluate beta and alpha diversities, we used the Quantitative Insights Into Microbial Ecology (QIIME) software package^18^. Linear discriminant analysis to identify taxa with differentiating abundance was conducted using the LDA Effect Size (LEfSe) algorithm^19^. Bacterial abundance was normalized by log-transformation, and statistical analysis and principal component analysis (clustering) were performed using the METAGENassist platform^20^.

### Euthanasia

Rats were anesthetized with CO_2_ and transcardially perfused with 0.1 M phosphate-buffered saline (PBS; pH 7.4) followed by 4% paraformaldehyde. Hindbrains and liver were harvested, postfixed in 4% paraformaldehyde for 2-h, and immersed in 30% sucrose, 0.1% NaN_3_ (Sigma-Aldrich; pH 7.4) in PBS and stored at 4 °C until processing.

### Microglia activation

Hindbrain samples were cryosectioned (Leica CM1950, Leica Biosystems, Wetzlar, Germany) at 20μm thickness. Sections were incubated overnight with a primary antibody against ionized calcium binding adaptor molecule 1 (Iba-1, Wako Cat#019-19741, RRDI: AB_839504) followed by Alexa-488 secondary antibody to visualize microglia activation as previously described^21^. Sections were mounted in ProLong (Molecular Probes, OR) and examined under a Nikon 80-I fluorescent microscope. The area fraction of Iba-1 was analyzed using Nikon Elements AR software as previously described^16,22^.

### Hepatic lipidosis

Liver samples were embedded in paraffin and cryosectioned at 4 µm thickness. Tissue sections were stained with hematoxylin and eosin (H&E). In addition, samples for Oil-Red-O (ORO) staining were embedded in optimum cutting temperature compound (VWR Inc., Atlanta, GA), cryosectioned at 7 µm thickness and stained with ORO (Polysciences Inc., Warrington, PA). H&E and ORO stained liver sections were examined microscopically buy a board-certified veterinary pathologist using an Olympus BX41 upright light microscope. Images were captured using an Olympus DP25 digital camera controlled by Olympus cellSense Standard software at ×200 and ×400 original magnification (Olympus, Shinjuku, Japan). A semi-quantitative grading scale (normal [0], minimal [1], mild [2], moderate [3], and marked [4]) was used to express the extent of hepatic lipidosis^23^. In addition, ORO staining was quantified by Nikon Elements AR Software as previously described^16^.

### Statistical analysis

GraphPad Prism 7 (GraphPad Software, Inc.) was used for statistical analyses. All samples were processed by an experimenter blind to the experimental condition. Data are expressed as mean ± SD and were analyzed using two-tailed *t*-test or ANOVA followed by Holm–Sidak multiple comparisons test as appropriate. Sample size was determined based on prior studies from our laboratory. To simplify statistical analysis and presentation of results, data for the ST-HED and LT-HED groups were analyzed independently. Alpha value was set at 0.05.

## Results

### HED diet significantly increased body weight and body fat mass

**Fig. 1:**
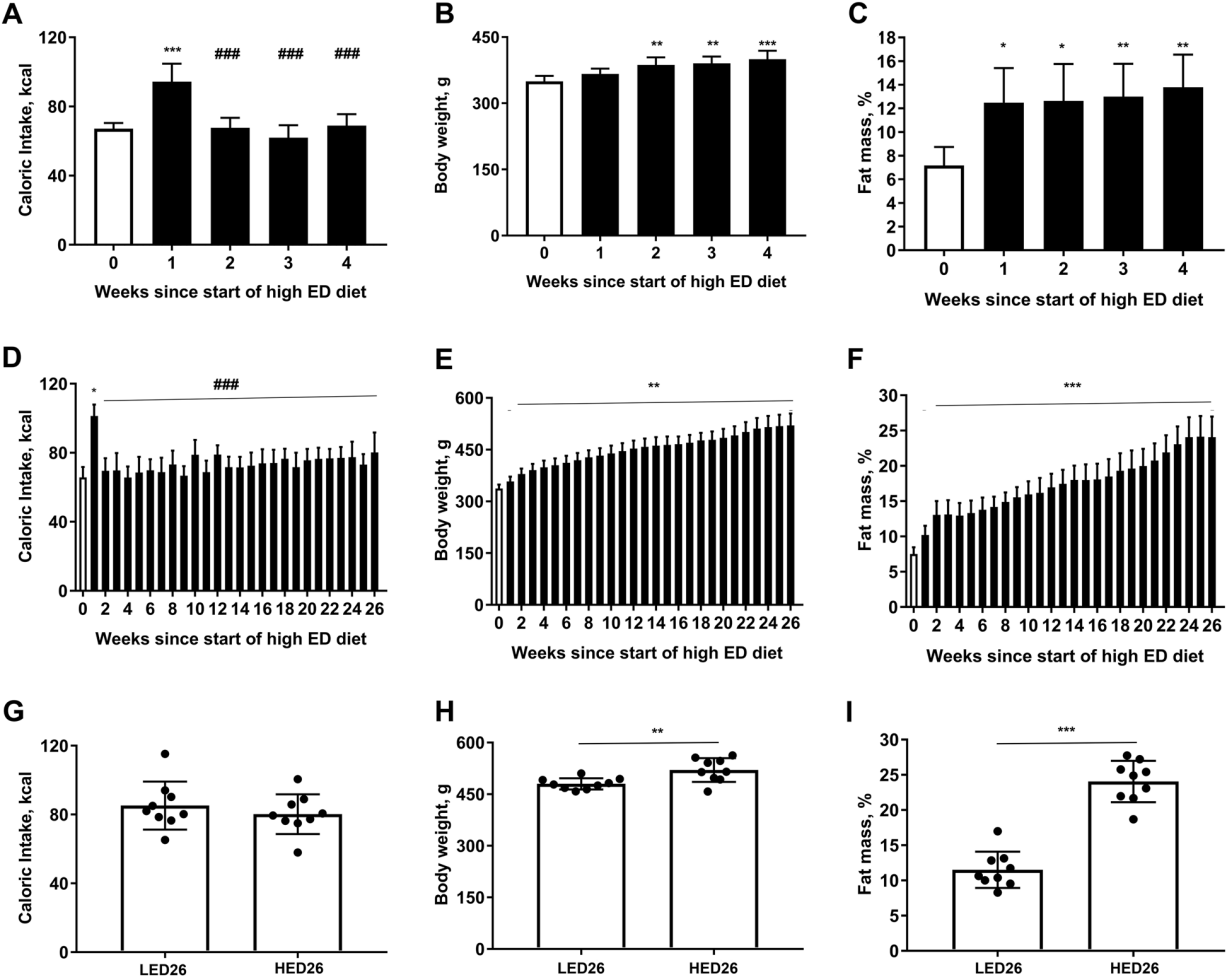
High energy density (HED) diet consumption significantly increased body weight and body fat mass. Shown are mean ± SD kcal consumed (**a**, **d**, **g**), body weight (**b**, **e**, **h**), and body fat mass (**c**, **f**, **i**) for rats fed HED for four weeks (ST-HED; *n* = 6, top row), rats fed HED for 26 weeks (LT-HED; *n* = 9, middle row), and endpoint comparison of rats fed LED vs HED for 26 weeks (*n* = 9 per group, bottom row). Animals significantly increased their caloric intake upon introduction of the HED diet, but caloric intake declined after 1 week and remained stable for the duration of the study in the ST-HED and LT-HED groups. HED diet consumption significantly increased body weight and fat mass. Asterisk indicates statistical significance from week 0. Hash indicates statistical significance from week 1 since start of HED diet, *P < 0.05, **P < 0.01, ***P < 0.0001.

Caloric intake, body weight, and body fat mass are shown in Fig. 1. In the ST-HED group, the animals significantly increased their caloric intake during the first week after introduction of the HED diet compared to intake of LED diet (Ps < 0.0001) (Fig. 1a, d). Caloric intake decreased to intakes of the LED by week 2 and remained stable. Body weight was significantly higher after one week of HED compared to baseline (Ps < 0.05) and the animals continued gaining weight (Fig. 1b, e). Body fat percent significantly increased after one week on HED diet compared to baseline (Ps < 0.05) and fat deposits continued to grow throughout the experiment (Fig. 1c, f). Caloric intake of the LT-HED group at the end of the study (HED26) was similar to that of aged-matched, LED diet controls (LED26) (Fig. 1g). However, body weight (*P* = 0.006) (Fig. 1h) and body fat (*P* < 0.0001) (Fig. 1i) were significantly higher in HED-fed rats compared to LED diet controls.

### HED diet triggered progressive dysbiosis of the gut microbiota

In the ST-HED group, the rarefaction curve of this analysis indicated >30,000 sequences and >500 OTUs per sample (Supplementary Fig. S2A). In the LT-HED group, the rarefaction curve of this analysis indicated >30,000 sequences and >1500 OTUs per sample (Supplementary Fig. S2B).

**Fig. 2:**
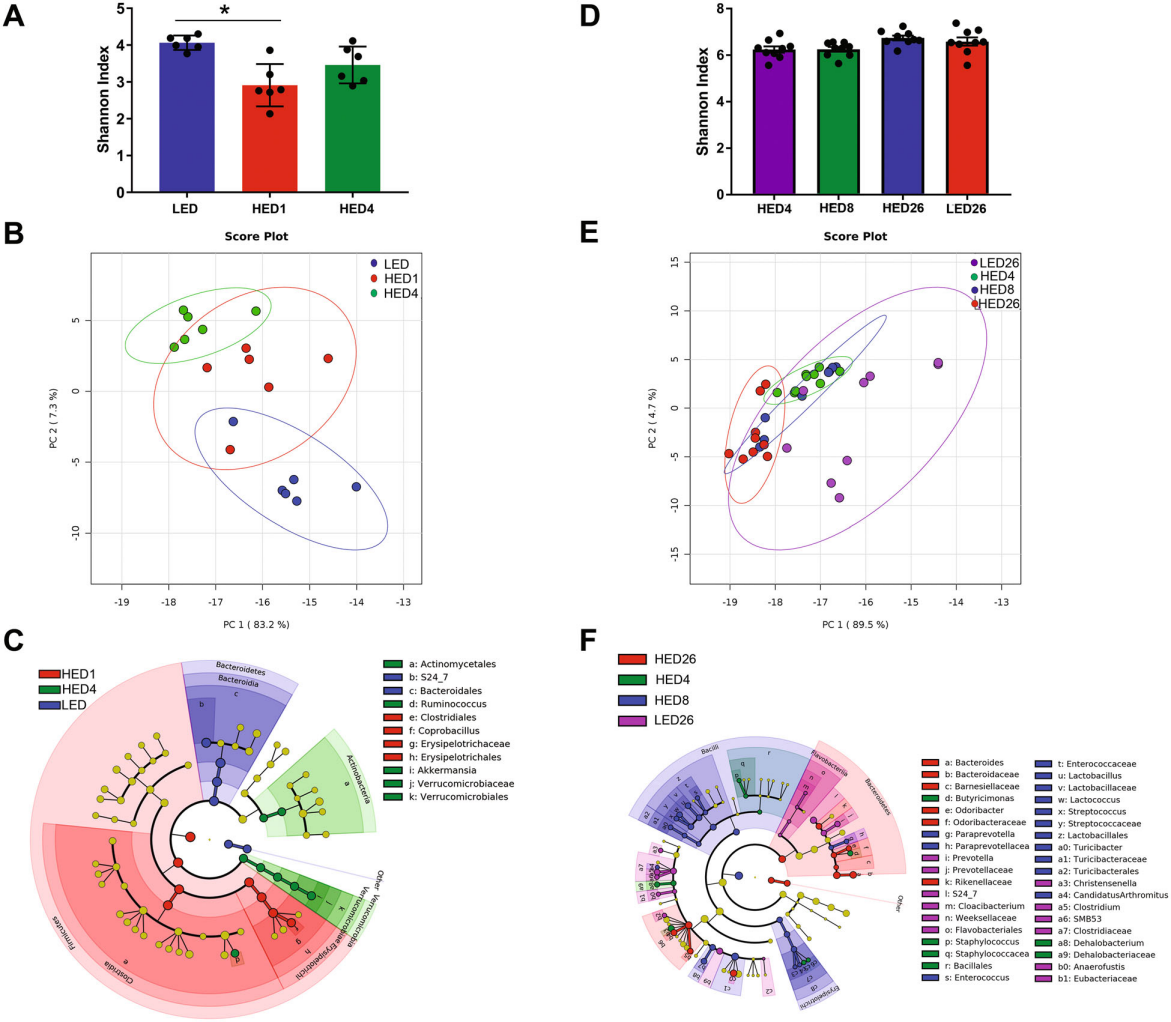
Consumption of a high energy density (HED) diet triggered progressive remodeling of the gut microbiota. The left (**a**, **b**, **c**) and right (**d**, **e**, **f**) columns represent data from the ST-HED (*n* = 6) and LT-HED (*n* = 9) groups, respectively. **a**, **d** Shannon index shown as mean ± SD for each group and time point. Bacterial diversity was significantly decreased after consuming the HED diet for one week (HED1) compared to baseline (LED). There were no other significant changes observed. **b**, **e** Principal Coordinate Analysis showing microbiota from all time points. In the ST-HED group (**b**), the microbiota of all rats clustered together at baseline (LED). One week after introduction of the HED diet (HED1), the microbiota clustered together and away from their LED profile. At week 4 (HED4), the microbiota clustered together and further away from their LED profile. In the LT-HED group (**e**), the microbiota of all rats fed the HED clustered together, independent of time point, and away from the microbiota of rats fed LED diet (LED26). **c**, **f** Cladogram produced from LDA scores (see Supplementary Fig. S2 for LDA scores). In the ST-HED group (**c**), at baseline (LED), the microbiota is characterized by abundant members of the *Bacteroidetes* order *Bacteroidales*. One week after HED diet introduction (HED1), the microbiota was characterized by abundant members of the *Firmicutes* order *Erysipelotrichales*. After 4 weeks of HED diet (HED4), the microbiota was characterized by abundant members of the *Actinobacteria* order *Actinomycetales* and *Verrucomicrobia* order *Verrucomicrobiales*. In the LT-HED group (**f**), at HED4 the microbiota was characterized by abundant members of *Bacteroidetes* order *Bacteroidales* and *Firmicutes* orders *Bacillales* and *Clostridiales*. Eight weeks after HED diet introduction (HED8), the microbiota was characterized by abundant members of the *Bacteroidetes* order *Bacteroidales* and *Firmicutes* orders *Lactobacillales, Turicibacterales*, and *Clostridiales*. After 26 weeks of HED diet (HED26), the microbiota was characterized by abundant members of *Bacteroidetes* orders *Bacteroidales*. The microbiota of the LED diet control group (LED26) was characterized by abundant members of *Bacteroidetes* orders *Bacteroidales* and *Flavobacteriales*, and *Firmicutes* order *Clostridiales*.

In the ST-HED and LT-HED groups, *Firmicutes* and *Bacteroidetes* were the most abundant phyla representing >90% of the bacteria identified. The Shannon index revealed a significant decrease in bacteria diversity after one week of HED diet compared to baseline (*P* = 0.0146) (Fig. 2a). There were no significant changes observed in bacterial diversity after 4 weeks. Principal Component Analysis (Fig. 2b) showed that at baseline (LED), all animals clustered together. One week after HED diet introduction, the animals clustered away from their baseline (LED) profile. After 4 weeks of HED diet, all animals clustered close to the profile at HED1 and further away from their LED profile.

Figure 2c (and Supplementary Fig. S3) represents the microbiota composition of the ST-HED group at each time point. At baseline (LED), the microbiota is characterized by abundant members of the *Bacteroidetes* order *Bacteroidales*. One week after HED diet introduction (HED1), the microbiota was characterized by abundant members of the *Firmicutes* order *Erysipelotrichales* and after 4 weeks by abundant members of the *Actinobacteria* order *Actinomycetales* and *Verrucomicrobia* order *Verrucomicrobiales*.

**Fig. 3:**
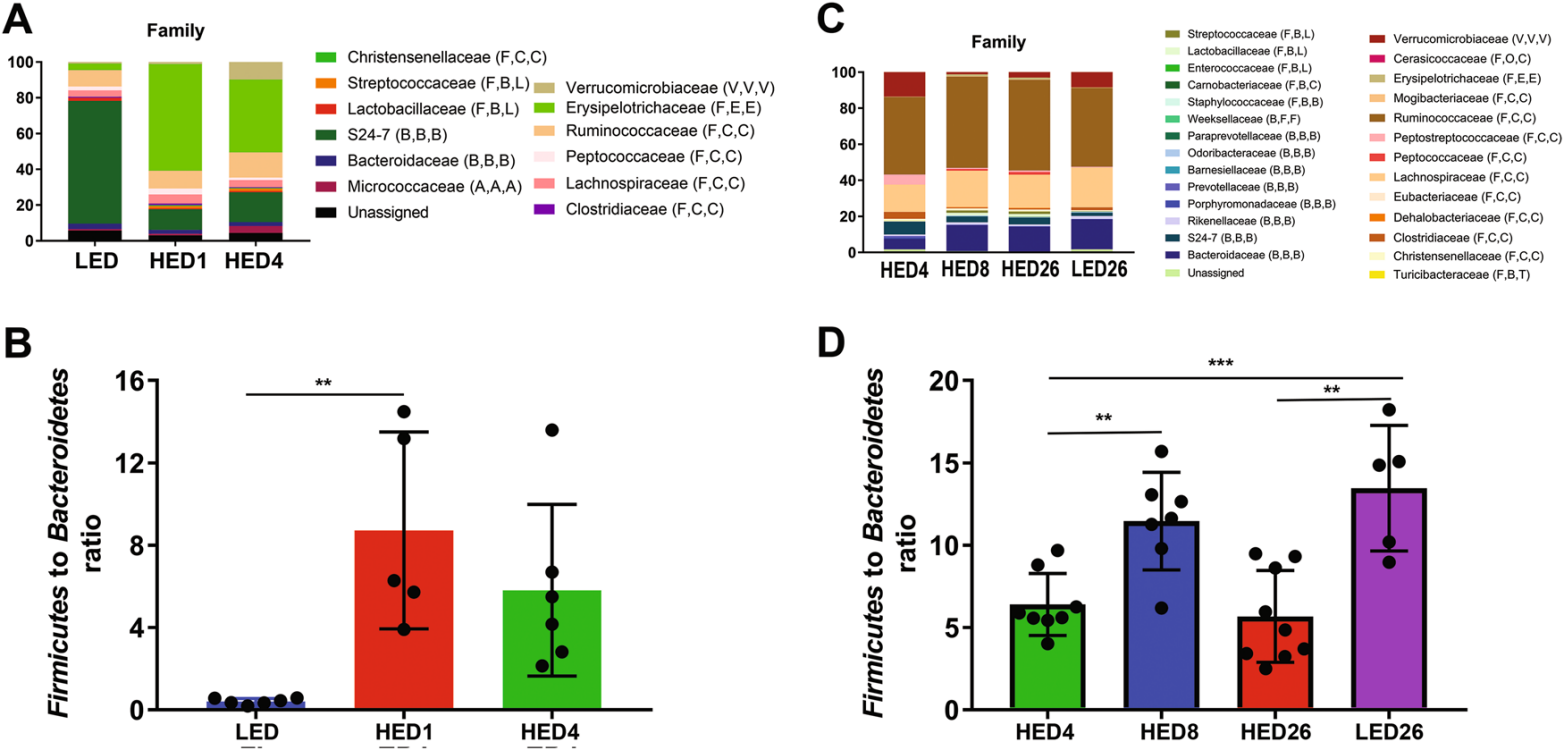
Microbial composition of rats fed a high energy density (HED) diet for 4 weeks (ST-HED, n = 6) or 26 weeks (LT-HED, *n* = 9) and rats fed a low energy density (LED) diet for 26 weeks (LF26, *n* = 9). All phylogenetic levels present with abundance >1% are represented. **a**, **c** relative abundances of phyla at the family level in the ST-HED group and in the LT-HED. **b**, **d** Ratio of Firmucutes to Bacteroidetes in the ST-HED and LT-HED group. In the ST-HED group, HED diet consumption significantly increased the abundance of members of *Erysipelotrichaceae* (LED 3.8% vs HED1 60% and HED4 41%, Ps < 0.0001) of the phylum *Firmicutes*. Members of the family *S24-7* of the phylum *Bacteroidetes* were significantly depleted by HED diet consumption (**a**). In addition, the ratio of *Firmicutes* to *Bacteroidetes* was significantly higher at 1 and 4 weeks of HED diet compared to LED (**b**). In the LT-HED group, compared to LED diet fed rats, HED diet fed rats had significantly higher abundance of members of *Bacteroidaceae* and *Ruminococcaceae*, and significantly lower abundance of members of *Peptostreptococcaceae* and *Verrucomicrobiaceae* (**c**). In HED-fed rats, the *Firmicutes-to-Bacteroidetes* ratio was significantly higher after eight weeks compared to after four and 26 weeks (HED8 11.5 vs HED4 6.4 and HED26 5.7, Ps < 0.01). Compared to LED controls (LED26), HED-fed rats had a significantly lower *Firmicutes-to-Bacteroidetes* ratio at four and 26 weeks (D). In the legend, following the name of each family, higher taxonomic classifications are indicated by letters in parentheses. Phylum: A, Actinobacteria; B, Bacteroidetes; F, Firmicutes; V, Verrucomicrobia. Class: A, Actinobacteria; B, Bacilli if preceded by F and Bacteroidia if preceded by B; C, Clostridia; E, Erysipelotrichia; F, Flavobacteria; V, Verrucomicrobiae; O, Opitutae. Order: A, Actinomycetales; B, Bacteroidales; C, Clostridiales if preceded by C and Cerasicoccales if preceded by O; E, Erysipelotrichiales; F, Flavobacteriales; L, Lactobacillales; T, Turibacterales; V, Verrucomicrobiales. Asterisk indicates statistical significance *P < 0.05, **P < 0.01, ***P < 0.0001. Data are means ± SD.

Microbiota composition changed within a week of introduction of HED diet (Fig. 3a). HED diet significantly increased the abundance of *Firmicutes* (LF 27% vs HED1 86% and HED4 69%, Ps < 0.0001) and decreased the abundance of *Bacteroidetes* (LED 67% vs HED1 10% and HED4 16%, Ps < 0.0001). There was also a significant increase in abundance of *Verrucomicrobia* after 4 weeks of HED diet (LED 1% and HED1 0.8% vs HED4 8%, *P* = 0.04). At the level of family, HED diet significantly increased the abundance of members of *Erysipelotrichaceae* (LED 3.8% vs HED1 60% and HED4 41%, Ps < 0.0001) of the phylum *Firmicutes*. Members of the family *S24-7* of the phylum *Bacteroidetes* were significantly depleted by HED diet (LED 69% vs HED1 12% and HED4 17%, Ps < 0.0001). In addition, 1 week of HED diet significantly increased the *Firmicutes-to-Bacteroidetes* ratio, (LED 0.4 vs HED1 8.7, *P* = 0.0010). There was no statistically significant difference after 4 weeks on the HED diet; however, the ratio was still higher at HED4 (4.3) than at LED (Fig. 3b).

In the LT-HED group, the Shannon index showed no significant difference in bacterial diversity (Fig. 2d). Principal Component Analysis showed that all animals fed the HED diet clustered together and away from animals fed LED (LED26) (Fig. 2e).

Figure 2f (and Supplementary Fig. S4) represents the microbiota composition of the LT-HED group at each time point. At HED4, the microbiota was characterized by abundant members of *Bacteroidetes* order *Bacteroidales* and *Firmicutes* orders *Bacillales* and *Clostridiales*, at HED8 by abundant members of the *Bacteroidetes* order *Bacteroidales* and *Firmicutes* orders *Lactobacillales, Turicibacterales*, and *Clostridiales*, and at HED26 by abundant members of *Bacteroidetes* orders *Bacteroidales*. The microbiota of the LED26 group was characterized by abundant members of *Bacteroidetes* orders *Bacteroidales* and *Flavobacteriales*, and *Firmicutes* order *Clostridiales*.

At the level of family (Fig. 3c), 4 weeks (HED4) and 8 weeks (HED8) after HED diet introduction there was a significantly higher abundance of *Ruminococcaceae* compared to after 26 weeks (HED26, *P* < 0.01) and a significantly lower abundance of *Verrucomicrobiaceae* compared to after 26 weeks (HED26, *P* < 0.01). HED diet fed rats had significantly higher abundance of members of *Bacteroidaceae* (LED26 0.8% vs HED4 14%, HED8 14%, and HED26 16%, Ps < 0.0001) and *Ruminococcaceae* (LED26 0.8% vs HED4 14% and HED8 14%, Ps < 0.0001) compared to LED fed rats. In addition, HED-diet-fed rats had significantly lower abundance of members of *Peptostreptococcaceae* (LED26 5% vs HED4 0.6%, HED8 0.9%, and HED26 0.5%, Ps < 0.05) and *Verrucomicrobiaceae* (LED26 14% vs HED4 1.2%, HED8 3%, and HED26 9%, Ps < 0.01) compared to LED fed rats. In HED-fed rats, the *Firmicutes-to-Bacteroidetes* ratio was significantly higher at HED8 compared to HED4 and HED26 (HED8 11.5 vs HED4 6.4 and HED26 5.7, Ps < 0.01). Compared to LED26 controls, HED-fed rats had a significantly lower *Firmicutes-to-Bacteroidetes* ratio at HED4 and HED26 (LED26 13% vs HED4 6% and HED26 6%, Ps < 0.01) (Fig. 3d).

### Long-term HED diet significantly changed the cytokine profile in serum

Mean (±SD) serum levels of cytokines (OD) and insulin (ng/ml) are shown in Table 1. In the ST-HED group, the data were compared using paired *t*-tests. We observed a significant increase in TNFα (*P* = 0.03) and significant decrease in IL-1α after four weeks on HED diet compared to baseline (*P* < 0.0001). No other significant changes were observed.

**Table 1:** Cytokine/chemokine optical density values ± SD.

	ST-HED		LT-HED				
	LED	HED4	LED	HED4	HED8	HED26	LED26
TNFα, OD	0.057 ± 0.016	0.044 ± 0.011^a^	0.049 ± 0.014	0.044 ± 0.006^b^	0.038 ± 0.003^a^	0.057 ± 0.016	0.062 ± 0.052
VEGF, OD	0.029 ± 0.006	0.026 ± 0.005	0.027 ± 0.005	0.025 ± 0.004	0.019 ± 0.002	0.024 ± 0.005	0.022 ± 0.011
FGFβ, OD	0.016 ± 0.001	0.015 ± 0.001	0.014 ± 0.003	0.028 ± 0.009^a c^	0.017 ± 0.007^c^	0.037 ± 0.007^a^	0.042 ± 0.042
IFNγ, OD	0.056 ± 0.019	0.045 ± 0.009	0.055 ± 0.015	0.036 ± 0.011	0.032 ± 0.005^a^	0.031 ± 0.004^a^	0.036 ± 0.031
Leptin, OD	0.049 ± 0.014	0.041 ± 0.005	0.042 ± 0.002 *n* = 8	0.068 ± 0.022^c^ *n* = 8	0.079 ± 0.071^c^ *n* = 8	0.472 ± 0.092^a d^ *n* = 8	0.089 ± 0.043 *n* = 8
MCP-1, OD	0.027 ± 0.007	0.022 ± 0.003	0.035 ± 0.007	0.08 ± 0.040^a c^	0.036 ± 0.027^c^	0.123 ± 0.039^a^	0.103 ± 0.075
SCF, OD	0.074 ± 0.024	0.077 ± 0.038	0.064 ± 0.021	0.058 ± 0.008^c^	0.053 ± 0.010^c^	0.124 ± 0.050^a d^	0.063 ± 0.032
MIP-1α, OD	0.019 ± 0.003	0.017 ± 0.006	0.014 ± 0.002	0.074 ± 0.043^a^	0.043 ± 0.046^c^	0.108 ± 0.019^a^	0.089 ± 0.043
IL-1α, OD	0.059 ± 0.005	0.027 ± 0.002^a^	0.054 ± 0.022	0.058 ± 0.044	0.032 ± 0.006^c^	0.067 ± 0.026^d^	0.024 ± 0.009
IL-1β, OD	0.069 ± 0.017	0.056 ± 0.005	0.059 ± 0.021	0.050 ± 0.009	0.043 ± 0.004	0.071 ± 0.028	0.054 ± 0.032
IL-5, OD	0.059 ± 0.018	0.047 ± 0.007	0.054 ± 0.016	0.032 ± 0.009	0.048 ± 0.049	0.029 ± 0.007^a^	0.029 ± 0.019
IL-6, OD	0.021 ± 0.003	0.021 ± 0.007	0.018 ± 0.003	0.022 ± 0.003	0.016 ± 0.003	0.031 ± 0.011	0.024 ± 0.012
IL-15, OD	0.051 ± 0.016	0.045 ± 0.010	0.046 ± 0.011	0.035 ± 0.005	0.031 ± 0.005^a c^	0.046 ± 0.012	0.042 ± 0.035
IP-10, OD	0.029 ± 0.004	0.032 ± 0.004	0.021 ± 0.007	0.021 ± 0.004^c^	0.026 ± 0.003	0.029 ± 0.004^a^	0.027 ± 0.007
Rantes, OD	1.183 ± 0.179	0.898 ± 0.169	1.191 ± 0.289	0.925 ± 0.317^c^	1.042 ± 0.312^c^	0.602 ± 0.207^a^	0.776 ± 0.252
TGFβ, OD	0.279 ± 0.065	0.187 ± 0.053	0.155 ± 0.012	0.091 ± 0.034^a^	0.198 ± 0.074	0.205 ± 0.099^d^	0.049 ± 0.009
Insulin, ng/ml	1.223 ± 0.187 *n* = 4	1.92 ± 0.346 *n* = 4	0.937 ± 0.298	1.055 ± 0.185	0.888 ± 0.181	1.133 ± 0.269	1.418 ± 0.486 *n* = 3

For ST-HED, LT-HED, and LED26 *n* = 6, 9, 9, respectively, unless otherwise stated.

^a^Different from LED.

^b^Different from HED8.

^c^Different from HED26.

^d^Different from LED26.

In the LT-HED group, 4 weeks after introduction of HED diet, we observed a significant increase in FGFβ, MCP-1, MIP-1a, and TGFβ compared to LED (Ps < 0.05). After 8 weeks of HED diet (HED8), IFNγ and IL-15 were significantly decreased compared to LED (Ps < 0.01). TNFα and IL-6 were significantly lower than after four weeks on HED diet (Ps < 0.05). After 26 weeks on HED diet (HED26), FGFβ, Leptin, SCF, and MCP-1 were significantly higher than at LED (Ps < 0.05), HED4 (Ps < 0.01), and HED8 (Ps < 0.05). IP-10 levels were significantly higher than at HED4 (*P* = 0.0094). MIP-1α, IL-15, and IL-1α were significantly higher than at HED8 (Ps < 0.05). Rantes was significantly lower compared to LED, HED4, and HED8 (Ps < 0.05). IFNγ, IL-5 levels were significantly lower compared to LED (Ps < 0.01). Cross-sectional comparison of the HED26 group to LED26 controls revealed that the HED26 group had significantly higher leptin, SCF, IL-1α, and TGFβ (Ps < 0.01).

### HED diet significantly increased microglia activation in the NTS

**Fig. 4:**
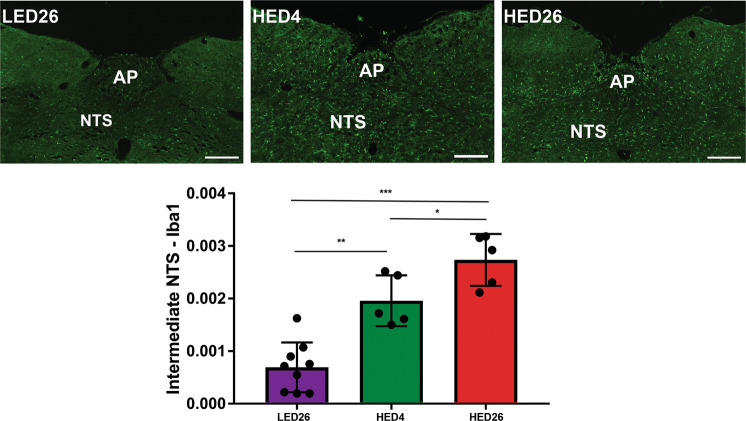
Consumption of a HED diet significantly increased microglia activation in the intermediate NTS. Representative sections of intermediate NTS of animals fed a LED diet for 26 weeks (LED26, *n* = 9), a HED diet for 4 weeks (HED4, *n* = 5), and a HED diet for 26 weeks (HED26, *n* = 5) are shown. Binary analysis of the area fraction of Iba-1 immunoreactivity showed that animals fed a HED for four and 26 weeks exhibited significantly more microglia activation than LED fed controls. In addition, microglia activation after 26 weeks of HED diet was significantly higher than after four weeks. Graphs represent mean ± SD Iba-1 intensity. Asterisk indicates statistical *P < 0.05, **P < 0.01, ***P < 0.0001. NTS nucleus tractus solitarius, AP area postrema. Scale bar = 200 μm.

Results of immunostaining against Iba-1 were compared using one-way ANOVA and revealed that compared to LED26 controls, rats fed HED diet for 4 weeks (HED4, *P* = 0.0005) and 26 weeks (HED26, *P* < 0.0001) had significantly higher binary area fraction of fluorescent staining against Iba-1 (Fig. 4). In addition, at HED26 binary area fraction was significantly higher than at HED4 (*P* = 0.0213).

### HED diet induced hepatic lipidosis

**Fig. 5:**
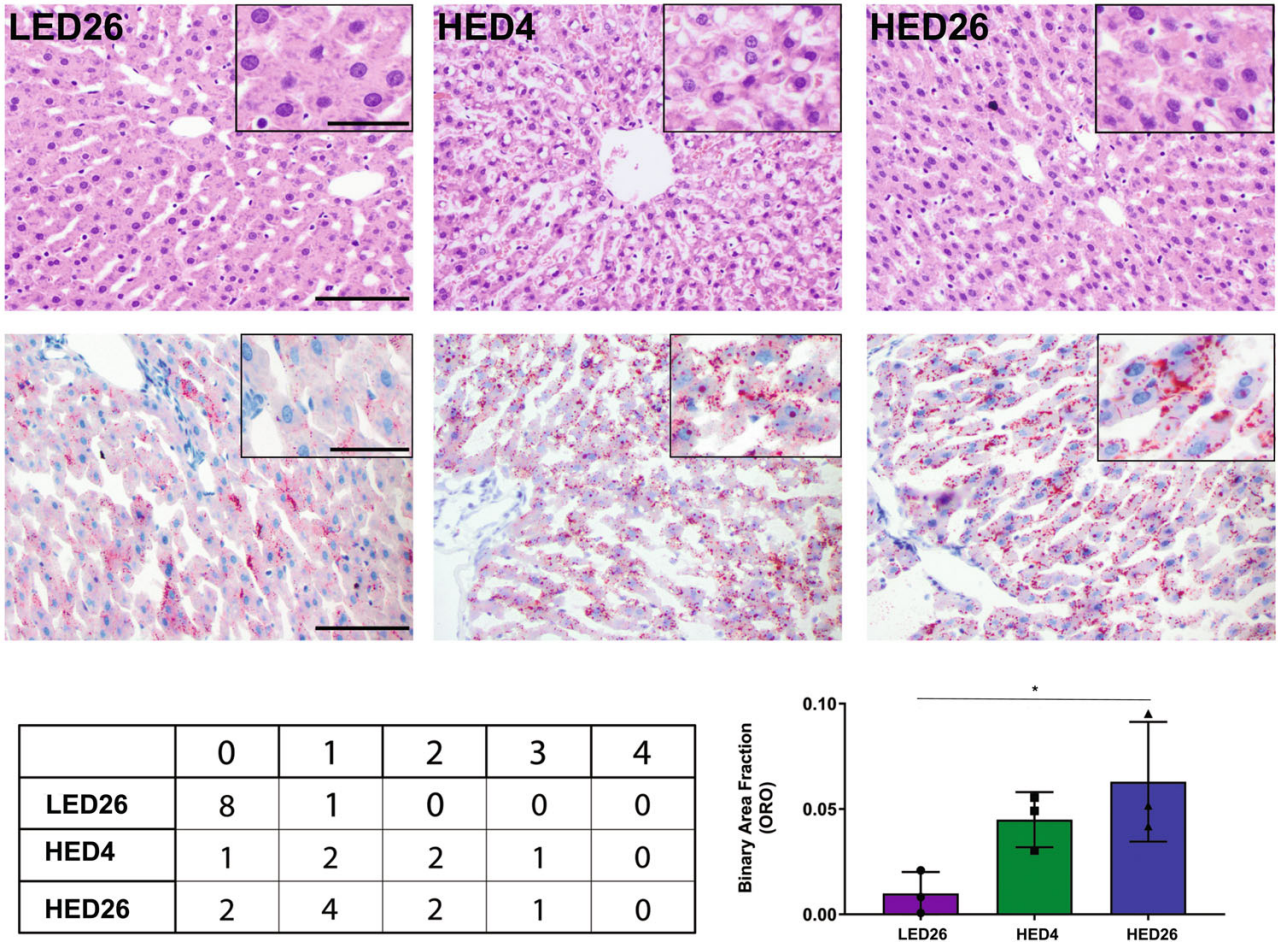
hED diet intake induces hepatic lipidosis. Representative histopathological images of hematoxylin and eosin stained (top row) and oil-red-o stained (bottom row) hepatic tissue from rats fed a LED diet (left column), rats fed a HED for 4 weeks (middle column), and rats fed a HED for 26 weeks (right column), *n* = 3 for each group. H&E staining revealed an increase in distinct vacuoles in rats fed the HED diet (HED4 and HED26) compared to LED controls (LED26) (top row). Similarly, ORO staining showed that HED-fed animals exhibited more intensely red granules (HED4 and HED26) than LED controls (LED26) (bottom row). The quantitative scoring (Table) confirmed that hepatocellular lipidosis is apparent after consuming a HED diet for four weeks. We did not observe significant differences in the extent of hepatocellular lipidosis between the ST-HED (HED4) and LT-HED (HED26) groups. LED fed rats did not show signs of hepatocellular lipidosis. Graph represents mean ± SD binary analysis of the area fraction of ORO staining, which further confirms our results. Asterisk indicates statistical significance *P < 0.05.

H&E (Fig. 5, top row) and ORO (Fig. 5, middle row) staining showed an increase in rats fed the HED diet (HED4 and HED26) compared to LED26 controls. The quantitative scoring confirmed that while animals fed LED diet (LED26) did not exhibit signs of hepatocellular lipidosis, it was apparent within 4 weeks of introducing the HED diet (HED4). There was no significant difference in hepatocellular lipidosis between the HED4 and HED26 time point. Software analysis revealed a progressive increase in lipid accumulation with LED fed animals showing very little staining (BAF = 0.0099 ± 0.01) compared to animals fed the HED diet for four weeks (BAF = 0.0449 ± 0.01) and 26 weeks (BAF = 0.0629 ± 0.02) (Fig. 5, bottom row table).

## Discussion

### HED diet significantly increased body weight and body fat mass

It is widely recognized that diet is a key factor in the composition, diversity, dynamics, and microbiota-driven host metabolism^24^. Consistent with prior reports^16,25,26^, rats fed HED diet did not altered their caloric intake long-term. We observed an initial increase, but the animals adjusted their intake within a few days^16,27^. In contrast, rats fed HED had significantly higher final body weight and fat mass compared to LED fed animals, despite similar caloric intake. This was previously reported in a study by Lomba et al., which showed that rats fed a high fat diet restricted to the amount of calories consumed by a low fat fed group gained significantly more body weight and white adipose tissue than the low fat fed group^28^. This phenomenon indicates that HED diets detrimentally affect body weight and fat mass accumulation independent of caloric intake in male Sprague–Dawley rats.

### HED diet triggered progressive dysbiosis of the gut microbiota

HED diet consumption induced dynamic fluctuations in the gut microbiota. Consistent with prior reports from our laboratory^25^, HED introduction triggered a rapid and transient decrease in bacterial diversity. It also induced marked fluctuations in bacterial abundance during the first 4 weeks. Thereafter, we did not observe further fluctuations in bacterial abundance.

We found that HED diet led to a rapid increase in members of the family *Erysipelotrichaceae* (*Firmicutes*). These are obligate anaerobes have been associated with consumption of HED, increased adiposity, and inflammation^29–31^. HED diet also depleted members of the families *S24-7* (*Bacteroidetes*) and *Verrucomicrobiaceae* (*Verrucomicrobia)*. Members of these families are associated with gut health as they are primarily involved in the fermentation of dietary fibers to produce short-chain fatty acids (SCFAs) and have been shown to be depleted by high fat diets^25,32–34^. After 4 weeks on the HED diet, we see a blooming of the family *Ruminococcaceae* (*Firmicutes*) that persists for the duration of the study. Members of this family are also SCFAs producers and generally associated with gut health^33^.

### Long-term HED diet consumption significantly changed the cytokine profile in serum

In summary, our results revealed that HED induced significant changes in FGFβ, IFNγ, leptin, MCP-1, SCF, MIP-1α, IL-1α, IL-5, IP-10, Rantes, and TGFβ.

Leptin was significantly higher only after 26 weeks on HED diet. Consistent with prior reports^35–37^, we did not observe changes in insulin. The effect of HED diet on insulin appears to be strain-specific. Studies have reported significant increase in plasma insulin in Long-Evans male rats fed a high fat diet for 70 days^38^, in WNIN rats after 13 weeks^37^, and Wistar rats after 18 weeks^39^.

Our results revealed a significant increase in MCP-1 and MIP-1α after 26 weeks of HED. A similar study by Muralidhar et al. reported no change in MIP-1α and a non-statistically significant increase in MCP-1 after 13 weeks of high fat feeding^37^. The length of time of HED diet likely underlies the differences observed between the two studies; however, there is an observable trend toward higher levels with HED diet consumption. In addition, we observed a significant decrease in Rantes, consistent with a prior report by Fenton et al. in mice fed a high fat diet for 10 weeks^40^.

SCF serves as a ligand molecule for the receptor tyrosine kinase c-Kit. Activation of c-Kit is involved in cell migration and survival^41^. Our results showed a significant increase in SCF after 26 weeks of HED diet consumption.

FGFβ is an endocrine hormone produced by the liver, which is thought to enhance insulin-mediated glucose uptake in the fed state^42^. IFNγ is an important cytokine for innate and adaptive immune response against viral infections. IP-10 is a chemokine produced in response to IFNγ that acts as a chemoattractant for a host of immune cells^43^. Our results showed a significant increase in FGFβ and IP-10, and a decrease and IFNγ after 26 weeks of HED diet consumption. However, when compared to aged-matched, LED fed rats, there were no significant differences. In Sprague–Dawley rats, Muralidhar et al. showed that a high fat diet for 13 weeks did not affect plasma levels of IP-10^44^. It is possible that the changes observed in this study are a result of the aging process and not triggered by the HED diet. In human subjects older than 50 years there is a decrease in IFNγ production from mononuclear cells^45^.

Interleukin 1α (IL-1α) is an epidermal proinflammatory cytokine^46^. Interleukin 5 (IL-5) is a key factor in the activation of eosinophils during allergic reactions^47^. Consistent with prior reports^40,48^, our data showed that HED diet fed rats had significantly higher levels of IL-1α and a significant decrease in IL-5 after 26 weeks.

### Consumption of a HED diet significantly increased microglia activation in the NTS

Reports from our laboratory and others have shown that consumption of a HED diet triggers microglia activation in the nodose ganglia, NTS, and hypothalamus^26,49,50^. Our data showed that HED diet induced an inflammatory response reflected by microglia activation in the intermediate NTS after 4 weeks. These data further suggest that length of exposure to the HED diet exacerbates this response since microglia activation after 26 weeks of HED diet was significantly higher than after four weeks.

### HED diet consumption induced hepatic lipidosis

Our results demonstrated that a HED diet significantly increased intracellular lipid accumulation in the liver. These data are in concert with prior studies in rodents, which report development of hepatic steatosis after 16 weeks of a 40% fat diet^51^. De Rudder et al., also reported that mice developed hepatic steatosis after only 4 weeks on a 60% fat diet^52^. Hepatic steatosis has been linked to microbiota dysbiosis^53,54^. The majority of the nutrient-rich blood supply to the liver comes from the intestines through the portal vein^55^. Thus, an increase in gut microbes that produce toxic/inflammatory byproducts increases the gut-derived bacterial products entering the liver^56^. Our data revealed an increase in abundance of members of the family *Erysipelotrichaceaea* and a study by Spencer et al., showed that levels of these bacteria are directly associated with changes in liver fat in female human subjects^57^. In addition, we saw an increase in SCFAs producers. The SCFAs acetate, propionate, and butyrate inhibit lipid accumulation in the liver and improve hepatic function in rodents^58–60^. Given that the abundance of *Ruminococcaceae* was increased after 26 weeks of HED, it is possible that the presence of these bacteria and their byproducts contributed to prevent the progression of hepatic steatosis, as there was no difference in the degree of steatosis after 4 weeks of HED

In conclusion, we showed that long-term consumption of a HED diet leads to increased adiposity, gut dysbiosis, hepatic steatosis, inflammation in the NTS, and increased systemic levels of inflammatory markers. This study is novel because, to our knowledge, it is the first to present longitudinal and cross-sectional results on the effect of long-term HED diets on all these parameters in a single cohort of animals. Our results suggests that gut dysbiosis starts immediately upon introduction of HED diet. As the liver is overloaded with accumulation of excess fat consumed, hepatic steatosis develops. At the same time, endotoxins produced by the resident gut microbiome damage vagal afferents, which in turn triggers microglia activation in the NTS. Then, cytokines are released from their production site (e.g., adipose tissue) into the systemic circulation. These responses are highly dynamic and play a significant role in the development of obesity.

## Supplementary information

Figure S1. Experimental design timeline.

Figure S2. Rarefraction curves by diet group and experimental time point. Data are shown as mean for rats fed a high energy density diet for 4 weeks (A, ST-HED) or 26 weeks (B, LT-HED).

Figure S3. LDA scores used for generation of cladogram (Fig. 3C). Colors designate time point: Blue: LED/baseline, Red: HED1, one week after introduction of HED diet, Green: HED4, four weeks after int

Figure S4. LDA scores used for generation of cladogram (Fig. 3F). Colors designate time point: Purple: LED26, after 26 weeks of LED diet, Green: HED4, four weeks after introduction of HED diet. Blue:

Supplementary Figure Legend
