# Supplementary figures and images for: Consumption of a high energy density diet triggers microbiota dysbiosis, hepatic lipidosis, and microglia activation in the nucleus of the solitary tract in rats
Source: Nutr Diabetes. 2020 Jun 9;10:20. doi: 10.1038/s41387-020-0119-4 (PMC7283362; doi:10.1038/s41387-020-0119-4)

**A)**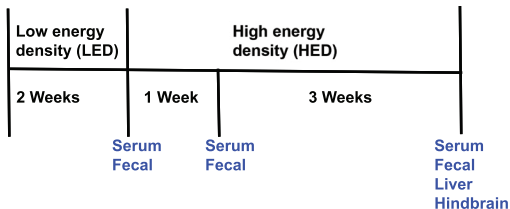**Short-term HED  
(ST-HED)****B)**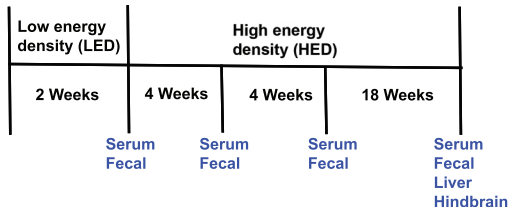**Long-term HED  
(LT-HED)****C)**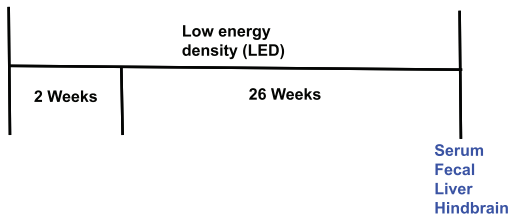**Long-term LED  
(LED26)**

Supplement: Supplementary file 1 — Figure S1. Experimental design timeline. [file 41387_2020_119_MOESM1_ESM.pdf]

**A**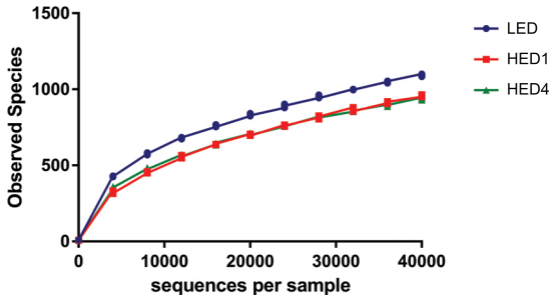**B**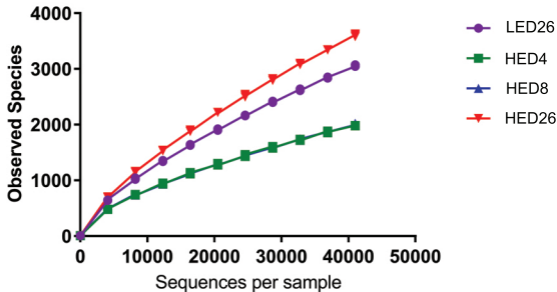

Supplement: Supplementary file 2 — Figure S2. Rarefraction curves by diet group and experimental time point. Data are shown as mean for rats fed a high energy density diet for 4 weeks (A, ST-HED) or 26 weeks (B, LT-HED). [file 41387_2020_119_MOESM2_ESM.pdf]

■ HED1 ■ HED4 ■ LED

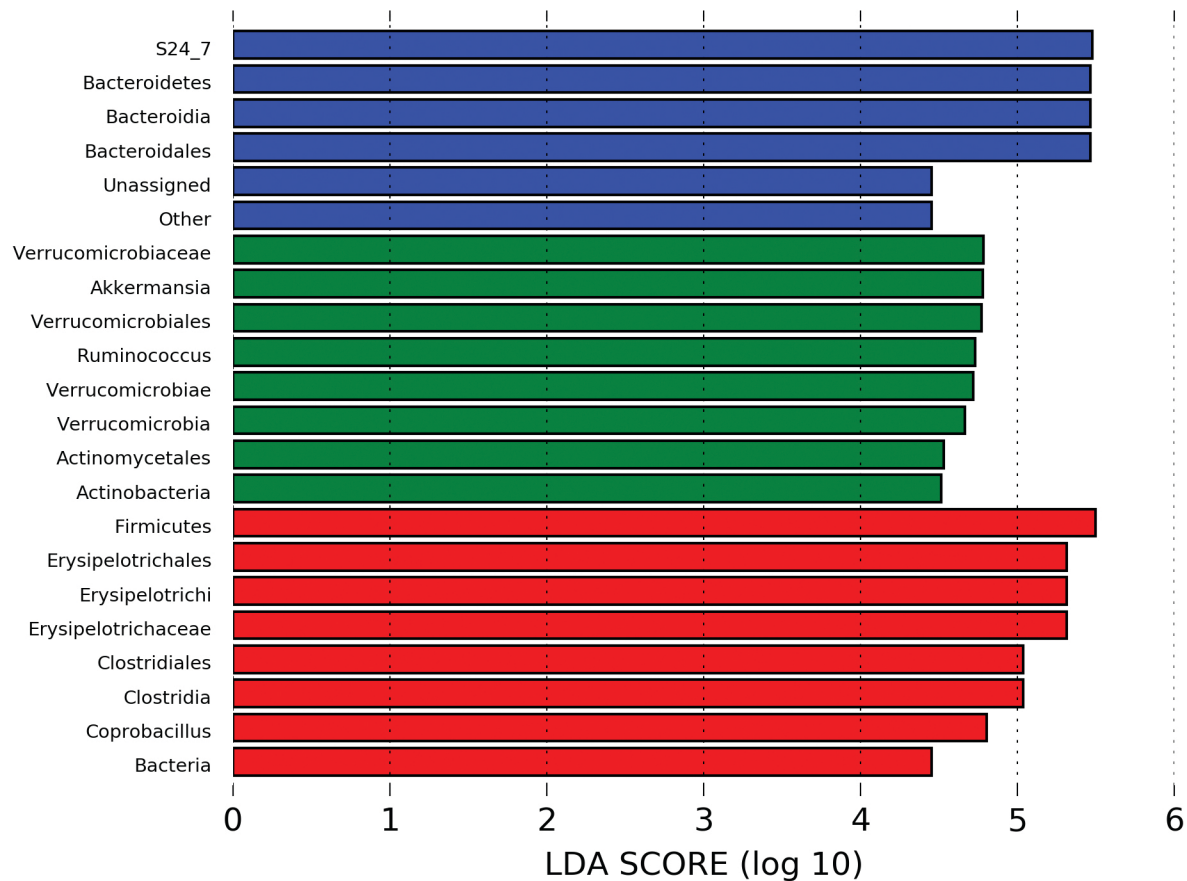

Supplement: Supplementary file 3 — Figure S3. LDA scores used for generation of cladogram (Fig. 3C). Colors designate time point: Blue: LED/baseline, Red: HED1, one week after introduction of HED diet, Green: HED4, four weeks after int [file 41387_2020_119_MOESM3_ESM.pdf]

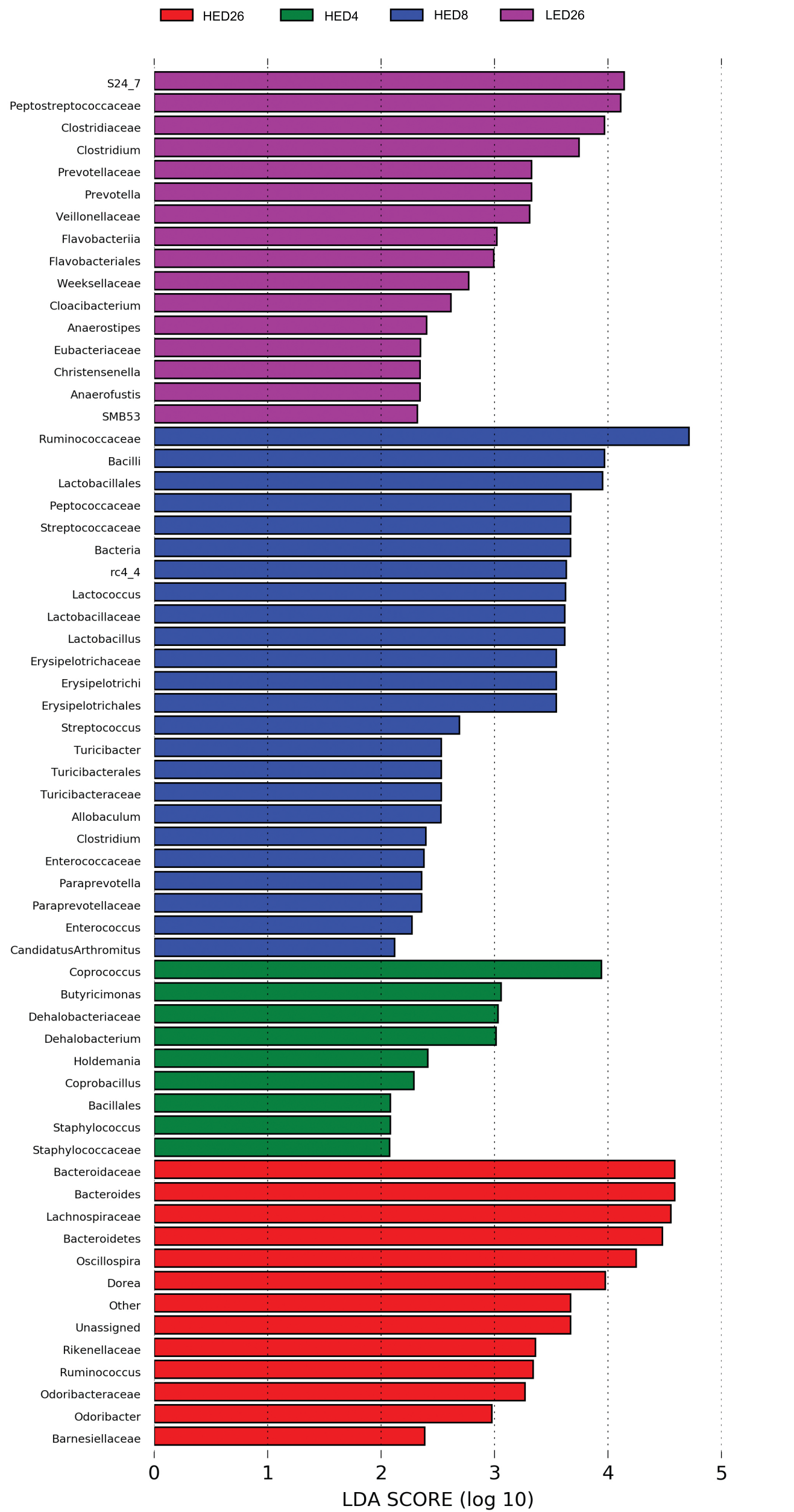

Supplement: Supplementary file 4 — Figure S4. LDA scores used for generation of cladogram (Fig. 3F). Colors designate time point: Purple: LED26, after 26 weeks of LED diet, Green: HED4, four weeks after introduction of HED diet. Blue: [file 41387_2020_119_MOESM4_ESM.pdf]
