# Supplementary material for: Consumption of a high energy density diet triggers microbiota dysbiosis, hepatic lipidosis, and microglia activation in the nucleus of the solitary tract in rats
Source: Nutr Diabetes. 2020 Jun 9;10:20. doi: 10.1038/s41387-020-0119-4 (PMC7283362; doi:10.1038/s41387-020-0119-4)
Supplement: Supplementary file 5 — Supplementary Figure Legend [file 41387_2020_119_MOESM5_ESM.docx]

**Supplementary Fig. S1.** Experimental design timeline.

**Supplementary Fig. S2.** Rarefraction curves by diet group and experimental time point. Data are shown as mean for rats fed a high energy density diet for 4 weeks (A, ST-HED) or 26 weeks (B, LT-HED).

**Supplementary Fig. S3.** LDA scores used for generation of cladogram (Fig. 3C). Colors designate time point: Blue: LED/baseline, Red: HED1, one week after introduction of HED diet, Green: HED4, four weeks after introduction of HED diet.

**Supplementary Fig. S4.** LDA scores used for generation of cladogram (Fig. 3F). Colors designate time point: Purple: LED26, after 26 weeks of LED diet, Green: HED4, four weeks after introduction of HED diet. Blue: HED8, four weeks after introduction of HED diet. Red: HED26, 26 weeks after introduction of HED diet.
